# Supplementary material for: Grant Review Feedback: Appropriateness and Usefulness
Source: Sci Eng Ethics. 2021 Mar 17;27(2):18. doi: 10.1007/s11948-021-00295-9 (PMC7969534; doi:10.1007/s11948-021-00295-9)
Supplement: Supplementary file 1 — Supplementary file1 (PDF 94 KB) [file 11948_2021_295_MOESM1_ESM.pdf]

Supporting Information

S1 File. Copy of Full Survey Text

Survey is listed below.
[]What is your gender?
Please choose all that apply:
Male
Female
Prefer not to answer
[]What is your age?
Please choose only one of the following:
Under 30
30–39
40–49
50–59
60+
[]Please specify your race/ethnicity
Please choose all that apply:
American Indian or Alaska Native
Asian or Asian American
Black or African American
Hawaiian or Other Pacific Islander
Hispanic or Latino
Non-Hispanic White/Caucasian
Other
Prefer not to answer
[]What type of degree(s) do you have?
Please choose all that apply:
PhD or other research doctorate
MD
DDS
DVM or VMD
Other
Prefer not to answer
[]What type of an organization do you work for?
Please choose only one of the following:
Academia
Government
Industry
Other
[]What stage of career have you reached?
Please choose only one of the following:
Early career
Mid career
Late career/tenured
Emeritus

[]On average, how many hours do you work each week?
Please choose only one of the following:
40 h
40–50 h
50–60 h
60–70 h
70 + h
[]Please provide any comments that justify your responses under Section 1, Demographics.
Please write your answer here:
Section 2: Grant submission and peer review experience
[]Have you submitted a grant for peer review in the last 3 years?
Please choose only one of the following:
Yes
No
[]If you answered yes to submitting a grant for peer review in the past 3 years, how many grant applications have you submitted in that time frame?
Please choose only one of the following:
1
2
3
4
5
6
7 or more
[]Did you receive reviewer feedback on your last grant submission?
Please choose only one of the following:
Yes
No
[]Was your last application successful, i.e., were you funded?
Please choose only one of the following:
Yes
No
[]Have you served on a peer review panel in the last 3 years?
Please choose only one of the following:
Yes
No
[]If you answered yes to serving on a peer review panel in the last 3 years, how many peer review panels have you served on in that time frame?
Please choose only one of the following:
1
2
3
4
5
6
7 or more

[]If you answered yes to serving on a peer review panel in the past 3 years, please select the mode of your last peer review panel meeting.
Please choose only one of the following:
Face-to-face
Remote (video/teleconference)
Internet-assisted
Other
[]How many ad-hoc reviews (usually one or two grant applications reviewed telephonically that are being evaluated in a panel meeting setting) have you performed in the past 3 years?
Please choose only one of the following:
0
1
2
3
4
5
6
7 or more
[]Have you reviewed for a journal in the last 3 years?
Please choose only one of the following:
Yes
No
[]If you answered yes to reviewing for a journal in the past 3 years, how many submissions have you reviewed in that time frame?
Please choose only one of the following:
1
2
3
4
5
6
7 or more
[]What is a higher personal priority: grant review or journal review?
Please choose only one of the following:
Grant review
Journal review
Both are equal priority
Neither is a priority
[]Please elaborate on your responses under Section 2, Grant submission and peer review experience.
Please write your answer here:
Section 3: Investigator attitudes toward grant review
If you answered yes to receiving feedback on your last grant submission, please answer Section 3 of the questionnaire. If you answered no, please proceed to Section 4.
[]On a scale of 1–5 (1 most useful, 5 least useful), overall how useful was the reviewer feedback you received on your last grant submission?

Please choose only one of the following:
1
2
3
4
5
[]On a scale of 1–5 (1 most useful, 5 least useful), how useful was the reviewer feedback in improving your grantsmanship?
Please choose only one of the following:
1
2
3
4
5
[]If you were not funded, on a scale of 1–5 (1 most useful, 5 least useful), how useful was the reviewer feedback in improving your future submissions?
Please choose only one of the following:
1
2
3
4
5
[]On a scale of 1–5 (1 most useful, 5 least useful), how useful was the reviewer feedback in informing your future scientific endeavors in the proposed research area?
Please choose only one of the following:
1
2
3
4
5
[]Did you feel the reviewer feedback was well written, cohesive, and balanced?
Please choose only one of the following:
Yes
No
[]Did you feel the reviewer feedback was fair and unbiased?
Please choose only one of the following:
Yes
No
[]Overall, in what area(s) did the reviewer feedback primarily focus?
Please choose all that apply:
Potential impact of research
Hypothesis
Research methodology
Innovation potential
Preliminary data
Responsiveness to funding mechanism

Statistical issues
Qualifications of research team
Budget
Other
[]Did the reviewers comment on the riskiness of the research project?
Please choose only one of the following:
Yes
No
[]Based on the reviewer feedback you received, do you feel that the reviewers had the appropriate expertise to evaluate your grant application?
Please choose only one of the following:
Yes
No
[]Please elaborate on your responses under Section 3, Investigator Attitudes Towards Grant review.
Please write your answer here:
Section 4: Reviewer attitudes towards grant review
[]What are your reasons for accepting an invitation to serve on a peer review panel?
Please choose all that apply:
Desire to give back to the scientific community
Networking opportunities
Informing your own grantsmanship
Gaining exposure to new and innovative scientific areas
Enhancing your career/resume
Expectation from the funding agency
Honorarium
Other
[]Do you feel that serving as a reviewer on peer review panels has positively impacted your career?
Please choose only one of the following:
Yes
No
[]If you feel that serving as a peer reviewer has positively impacted your career, in what ways has serving as a reviewer influenced your career?
Please choose all that apply:
Bolstered your career
Improved your grantsmanship
Increased your exposure to new scientific ideas
Improved your networking/collaboration opportunities
Other
[]In general, which type of panel meeting format do you prefer?
Please choose only one of the following:
Face-to-face
Virtual [teleconference/videoconference]
Internet-assisted

[]On a scale of 1–5, (1 most influential, 5 least influential), please rate the following factors in influencing your selection of preferred panel meeting format:
Please write your answer(s) here:
Logistical convenience
Level of communication among panel members
Networking opportunities
Likelihood to participate on panel
[]In the last 3 years, how many times have you declined an invitation to serve on a peer review panel?
Please choose only one of the following:
1
2
3
4
5
6
7 or more
[]What were your reasons for declining an invitation to serve on a peer review panel?
Please choose all that apply:
Limited free time
Poor expertise match
Personal reasons (holiday, sickness, travel)
Review timeline too compressed
Conflict of interest
Issue with funding agency
Other
[]What is the maximum number of peer review panels/committees you prefer to serve on per year?
Please choose only one of the following:
1
2
3
More than 3
[]What is the maximum number of days you prefer to attend a peer review panel meeting?
Please choose only one of the following:
1
2
3
More than 3
[]What is the maximum number of R01-type grant applications you prefer to be assigned for a peer review panel meeting?
Please choose only one of the following:
1–2
3–4
5–6
7

More than 7
[]What was the actual number of days of your last peer review panel meeting?
Please choose only one of the following:
1
2
3
More than 3
[]What was the actual number of R01-type grant applications you were assigned to review at your last peer review panel meeting?
Please choose only one of the following:
1–2
3–4
5–6
7–8
More than 8
[]On average, how many hours did you spend reviewing each grant application before the panel meeting?
Please choose only one of the following:
1–2
2–3
3–4
4–5
5–6
7 or more
[]Please elaborate on your responses under Sect. 4, Reviewer attitudes towards grant review.
Please write your answer here:
Section 5: Peer review panel meeting proceedings
[]Please answer the following questions in relation to your last peer review meeting. On a scale of 1–5 (1 most definitely, 5 not at all), was your scientific expertise necessary and appropriately used in the review process?
Please choose only one of the following:
1
2
3
4
5
[]On a scale of 1–5 (1 most definitely, 5 not at all), from your perspective was the expertise of the other panel members necessary and appropriately used in the review process?
Please choose only one of the following:
1
2
3
4
5
[]Did the grant application discussions facilitate reviewer participation?
Please choose only one of the following:

Yes
No
[]Were the grant application discussions fair and balanced?
Please choose only one of the following:
Yes
No
[]On a scale of 1–5 (1 most useful, 5 least useful), how useful were the grant application discussions in clarifying differing reviewer opinions?
Please choose only one of the following:
1
2
3
4
5
[]On a scale of 1–5 (1 extremely effective, 5 no effect), did the grant application discussions affect the outcome?
Please choose only one of the following:
1
2
3
4
5
[]On a scale of 1–5 (1 most appropriate, 5 least appropriate), were the evaluation criteria appropriate to judge the best science and move the field forward?
Please choose only one of the following:
1
2
3
4
5
[]On a scale of 1–5 (1 extremely important, 5 of no importance), how important is the PI's track record to assessing an investigator initiated (R01)-type application?
Please choose only one of the following:
1
2
3
4
5
[]In general, does a PI's track record temper your assessment of any detected methodological weaknesses?
Please choose only one of the following:
Yes
No
[]On a scale of 1–5 (1 most definitely, 5 not at all), did the grant application discussions promote the best science?
Please choose only one of the following:

1
2
3
4
5
[]Was innovation factored into selecting the best science?
Please choose only one of the following:
Yes
No
[]Did you view innovation as an essential component of scientific excellence when evaluating the grant applications?
Please choose only one of the following:
Yes
No
[]Did the risks associated with innovative research impact the scores you assigned to the grant applications?
Please choose only one of the following:
Yes
No
[]On a scale of 1–5 (1 completely, 5 not at all), how much did the seniority of your fellow panel members influence your evaluations during the panel deliberations?
Please choose only one of the following:
1
2
3
4
5
[]Was the format and duration of the grant application discussions sufficient to allow the non-assigned reviewers to cast well informed merit scores?
Please choose only one of the following:
Yes
No
[]On a scale of 1–5 (1 extremely useful, 5 not useful at all), how useful was the Chair in facilitating the application discussions?
Please choose only one of the following:
1
2
3
4
5
[]Please elaborate on your responses under Section 5, Peer review panel meeting proceedings.
Please write your answer here:
Thank you for taking the time to fill out the survey. Have a wonderful day!
Submit your survey.
Thank you for completing this survey.
