# Supplementary material for: Grant Review Feedback: Appropriateness and Usefulness
Source: Sci Eng Ethics. 2021 Mar 17;27(2):18. doi: 10.1007/s11948-021-00295-9 (PMC7969534; doi:10.1007/s11948-021-00295-9)
Supplement: Supplementary file 2 — Supplementary file2 (DOCX 20 KB) [file 11948_2021_295_MOESM2_ESM.docx]

**Appendix**

**Supplementary Table 1** – Respondent Comments Grouped by Text Presence of Keyword Relevant to Survey Question

| Keyword category (Relevant Survey Question) | % of Total Grouped Comments (N) | % Positive Only | %Negative Only | %Both Pos/Neg |
| --- | --- | --- | --- | --- |
| Written  *Q1: Did you feel the reviewer feedback was well written, cohesive, and balanced?* | 10% (8) | 0% | 38% | 63% |
| Bias  *Q2: Did you feel the reviewer feedback was fair and unbiased?* | 33% (26) | 0% | 54% | 46% |
| Expertise  *Q3: Based on the reviewer feedback you received, do you feel that the reviewers had the appropriate expertise to evaluate your grant application?* | 48% (38) | 0% | 39% | 61% |
| Useful/Helpful  *Q4. On a scale of 1-5 (1 most useful, 5 least useful), overall how useful was the reviewer feedback you received on your last grant submission?*  *Q5. On a scale of 1-5 (1 most useful, 5 least useful), how useful was the reviewer feedback in improving your grantsmanship?*  *Q6. If you were not funded, on a scale of 1-5 (1 most useful, 5 least useful), how useful was the reviewer feedback in improving your future submissions?*  *Q7. On a scale of 1-5 (1 most useful, 5 least useful), how useful was the reviewer feedback in informing your future scientific endeavors in the proposed research area?* | 33% (26) | 8% | 31% | 62% |

Proportions calculated based on a total of 79 comments

**Supplementary Table 2** – Comparisons of the quantitative answers to questions for Usefulness and Appropriateness for two waves of responses (first wave versus second wave).

| Survey Question | First Wave:  %Yes (95%CI) or Median ± 95%CI | Second Wave: %Yes (95%CI) or Median ± 95%CI |
| --- | --- | --- |
| *Q1: Did you feel the reviewer feedback was well written, cohesive, and balanced?* | 55% (50% to 60%) | 57% (51% to 63%) |
| *Q2: Did you feel the reviewer feedback was fair and unbiased?* | 62% (56% to 68%) | 57% (51% to 63%) |
| *Q3: Based on the reviewer feedback you received, do you feel that the reviewers had the appropriate expertise to evaluate your grant application?* | 59% (54% to 64%) | 57% (51% to 63%) |
| *Q4. On a scale of 1-5 (1 most useful, 5 least useful), overall how useful was the reviewer feedback you received on your last grant submission?* | 3.0 ± 0.12 | 3.0 ± 0.14 |
| *Q5. On a scale of 1-5 (1 most useful, 5 least useful), how useful was the reviewer feedback in improving your grantsmanship?* | 3.0 ± 0.12 | 3.0 ± 0.15 |
| *Q6. If you were not funded, on a scale of 1-5 (1 most useful, 5 least useful), how useful was the reviewer feedback in improving your future submissions?* | 3.0 ± 0.14 | 3.0 ± 0.16 |
| *Q7. On a scale of 1-5 (1 most useful, 5 least useful), how useful was the reviewer feedback in informing your future scientific endeavors in the proposed research area?* | 3.0 ± 0.13 | 3.0 ± 0.15 |

**Supplementary Table 3** – Demographics and Respondents with Comments

|  |  | Proportion That Made a Comment (n) [Total N =216] | Proportion That Did Not Make a Comment (n) [Total N =417] |
| --- | --- | --- | --- |
| Gender | Men | 62% (133) | 64% (265) |
|  | Women | 38% (83) | 36% (152) |
|  |  |  |  |
| Age | Under 50 | 21% (44) | 32% (132) |
|  | 50 and Over | 79% (169) | 68% (278) |
|  |  |  |  |
| Race/Ethnicity | White | 83% (180) | 71% (297) |
|  | Non-White | 17% (36) | 29% (120) |
|  |  |  |  |
| Degree Type | PhD | 84% (182) | 82% (342) |
|  | Non-PhD | 16% (34) | 18% (75) |
|  |  |  |  |
| Organization | Academia | 88% (188) | 88% (364) |
|  | Non-Academia | 12% (25) | 11% (46) |
|  |  |  |  |
| Career Stage | Early/Mid | 27% (57) | 38% (156) |
|  | Late/Tenured/Emeritus | 73% (156) | 62% (256) |

**Supplementary Table 4** – Example Respondent Comments Grouped by Survey Question

| Relevant Survey Question | Number | Comment |
| --- | --- | --- |
| *Q1: (well written, cohesive, and balanced?)* | Q1.1 | *“Current reviews are very short and written as bullet points. Therefore, there is normally criticism but no guidance which makes the review absolutely not useful for future improvement/applications.”* |
|  | Q1.2 | *“The critiques seemed to be hastily written (as they are, from my live-panel reviewer experience). Most annoyingly, NIH reviewers often down-score a proposal without comment, although again from my experience as a reviewer, I know they are instructed NOT to do that. In such cases they are in essence telling me that there was something they didn't like, but couldn't or wouldn't say what it was. This makes the information un-actionable, and thus useless.”* |
| *Q2: (fair and unbiased?)* | Q2.1 | “*Reviewers these days are often quite biased towards specific methodologies, often the ones they use*.” |
|  | Q2.2 | “*There is too much personal bias in grant review. Reviewers seem to have the people they want to champion and shoot down others they do not know.”* |
|  | Q2.3 | *“I believe there is too much in the way of politics and also bias against women in the peer review process.”* |
|  | Q2.4 | “*It takes just one biased or not knowledgeable reviewer to sink a grant application*.” |
| *Q3: (appropriate expertise?)* | Q3.1 | *“I feel that reviewers often lack technical background in my field and instead substitute comments related to empirical details that should be less important. But some reviewers do possess the necessary technical background.”* |
|  | Q3.2 | *“The last grant submitted was a mix of oncology, nutrition and immunology. No one reviewer could cover all of this expertise.”* |
| *Q4-7: (useful reviewer feedback?)* | Q4-7.1 | *“The last review was very short, but it did touch on some very important questions about the feasibility of completing the project. It is worth adding that this was a project that was previously submitted twice before to different organizations. Each time, the reviews were incredibly helpful and I believe they led me to improve the proposal during each iteration to the point it was eventually funded... which is the whole point of the peer review... constructive criticism.”* |
|  | Q4-7.2 | *“The application scored on the verge of fundability, this score had clearly been lowered by people who had voted outside the reviewer’s limits. The resubmission, incorporating improvements suggested by the original review, was triaged. Such inconsistency between panels implies a profoundly unreliable quality of review and tends to confirm the view that we are in a casino rather than an effectively regulated system.”* |
|  | Q4-7.3 | *“While reviewers have often been helpful, they are frequently less helpful when they make sweeping comments outside their area of expertise, especially statistics – where they sometimes criticize methods they are unfamiliar with or don’t understand, or worse-yet, ask for irrelevant/inappropriate analyses to be added.”* |
|  | Q4-7.4 | *“The reviews were one sentence ‘bullets’ (these were NIH applications). NIH has (in my experience as a reviewer within the past decade) given reviewers explicit instructions to NOT offer suggestions for alternative approaches – and the reviews we received stated only that ‘X was not likely to be useful / informative / etc.’.”* |
|  | Q4-7.5 | *“The reviews are often helpful, but frequently they are also misfires. By that, I mean they bring up some issue that nobody else has ever brought up, and something we will never see again. If we transform our proposal in response to such unusual comments, we will be bouncing around too much. Yet such a comment can kill a grant. There is far too much noise in the system. Also, the ability to distinguish good grants from excellent ones is diminished because of such noise. Say that adds +/-10% error into the system. Yet the pay lines are sometimes at this level of noise!”* |

**Supplementary Table 5** - Answers for Appropriateness Questions and Respondents with Comments

| Question (Appropriateness) | Commenting Respondents | | Non-Commenting Respondents | | Comparison |
| --- | --- | --- | --- | --- | --- |
|  | % Yes | Total N | % Yes | Total N | Chi-square |
| Q1: Did you feel the reviewer feedback was well written, cohesive, and balanced? | 45% | 207 | 61% | 405 | X^2^ [1]=14.7, p=0.0001, phi=0.16 |
| Q2: Did you feel the reviewer feedback was fair and unbiased? | 44% | 202 | 69% | 392 | X^2^ [1]=34.3, p<0.0001, phi=0.24 |
| Q3: Based on the reviewer feedback you received, do you feel that the reviewers had the appropriate expertise to evaluate your grant application? | 44% | 203 | 66% | 393 | X^2^ [1]=25.0, p<0.0001, phi=0.20 |

** indicates p<0.01

**Supplementary Table 6** – Variance Inflation Factors (VIFs) for independent variables from the logistic regression models for Q1-7

| Factor | Men | Age  (Over 50)? | White? | PhD? | Academia? | Early /  Mid-Career? | Funded? |
| --- | --- | --- | --- | --- | --- | --- | --- |
| VIF – Q1  (Well Written) | 1.02 | 1.29 | 1.04 | 1.02 | 1.02 | 1.33 | 1.01 |
| VIF – Q2  (Fair/Unbiased) | 1.02 | 1.29 | 1.03 | 1.02 | 1.02 | 1.31 | 1.01 |
| VIF – Q3  (Expertise) | 1.02 | 1.34 | 1.04 | 1.03 | 1.03 | 1.37 | 1.01 |
| VIF – Q4  (Overall Useful) | 1.03 | 1.33 | 1.03 | 1.03 | 1.03 | 1.36 | 1.01 |
| VIF – Q4  (Grantsmanship-Useful) | 1.03 | 1.35 | 1.05 | 1.03 | 1.04 | 1.39 | 1.01 |
| VIF – Q4  (Future Submissions-Useful) | 1.04 | 1.32 | 1.05 | 1.03 | 1.03 | 1.33 | 1.02 |
| VIF – Q4  (Future Research Endeavors-Useful) | 1.03 | 1.30 | 1.05 | 1.02 | 1.04 | 1.34 | 1.01 |
